# Supplementary material for: Gle1 is required for tRNA to stimulate Dbp5 ATPase activity in vitro and to promote Dbp5 mediated tRNA export in vivo
Source: bioRxiv. 2023 Oct 10:2023.06.29.547072. Originally published 2023 Jun 29. Preprint. [Version 3] doi: 10.1101/2023.06.29.547072 (PMC10327206; doi:10.1101/2023.06.29.547072)
Supplement: Supplementary file 1 [file NIHPP2023.06.29.547072v3-supplement-2.pdf]

bioRxiv preprint doi: https://doi.org/10.1101/2023.06.29.547072; this version posted October 10, 2023. The copyright holder for this preprint (which was not certified by peer review) is the author/funder, who has granted bioRxiv a license to display the preprint in perpetuity. It is made available under aCC-BY 4.0 International license.

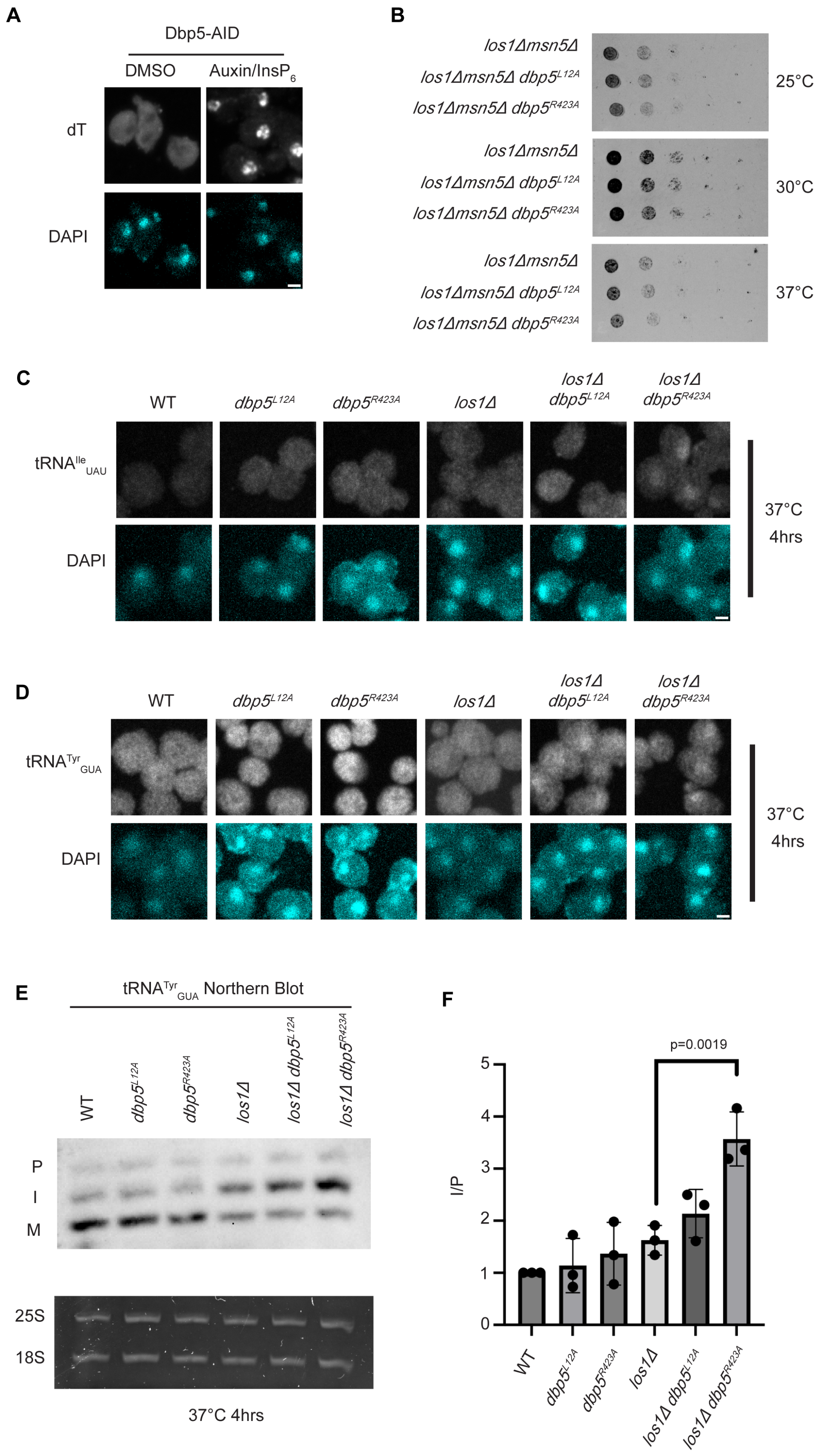

bioRxiv preprint doi: https://doi.org/10.1101/2023.06.29.547072; this version posted October 10, 2023. The copyright holder for this preprint (which was not certified by peer review) is the author/funder, who has granted bioRxiv a license to display the preprint in perpetuity. It is made available under aCC-BY 4.0 International license.

Figure 1 Supplement: Dbp5 functions parallel to Los1 in pre-tRNA export

(A) dT FISH confirms induction of mRNA export defect and Dbp5 loss of function after addition of 500 □M Auxin and 10 □M InsP$_6$ in Dbp5-AID strain for 90 minutes. Scale bar represents 2 μm.

(B) Spot assay for growth of shuffle strains containing untagged *dbp5*^{L12A} or *dbp5*^{R423A} on Leu marked CEN plasmids in combination with *los1Δ/msn5Δ.* Genomic copy of Dbp5 has been replaced with His6Mx marker. Growth for two days at 25, 30, and 37°C on YPD was conducted following two rounds of counter selection for Ura marked WT Dbp5 CEN plasmids on 5'FOA.

(C) tRNA FISH using a probe that can hybridize to the intron-containing and spliced isoform of tRNA$^{Ile}_{UAU}$ in indicated strains after pre-culture to early log phase at 25°C and shift to 37°C for 4 hours. Scale bar represents 2 μm.

(D) tRNA FISH using a probe that can hybridize to the intron-containing and spliced isoform of tRNA$^{Tyr}_{GUA}$ in indicated strains after pre-culture to early log phase at 25°C and shift to 37°C for 4 hours. Scale bar represents 2 μm.

(E) Northern Blot analysis targeting precursor and mature isoforms of tRNA$^{Tyr}_{GUA}$. Small RNAs were isolated from strains at mid log phase growth after pre-culture at 25°C and shift to 37°C for 4 hours. "P" bands represent intron containing precursors that have 5' leader/3' trailer sequences and "I" bands represent intron-containing end processed tRNA intermediates that have leader/trailer sequences removed.

(F) Quantification of Northern blot from (E). Ratio of signal from intron-containing end processed intermediates (I) vs 5' leader/3' trailer containing precursor (P) was calculated and presented relative to I/P ratio observed for WT. Error bars represent standard deviation and p-values calculated using one-way ANOVA.
